# Cardiomiopatia Chagásica Crônica: Influência do Exercício Físico

**DOI:** 10.36660/abc.20240507

**Published:** 2024-09-18

**Authors:** Gustavo Augusto Ferreira Mota, Mariana Gatto, Vitor Loureiro da Silva, Paula Felippe Martinez, Marina Politi Okoshi

**Affiliations:** 1 Universidade Estadual Paulista Faculdade de Medicina de Botucatu Departamento de Clínica Médica São Paulo SP Brasil Departamento de Clínica Médica – Faculdade de Medicina de Botucatu – Universidade Estadual Paulista (UNESP), São Paulo, SP – Brasil; 2 Universidade Federal de Mato Grosso do Sul Instituto Integrado de Saúde Campo Grande MS Brasil Instituto Integrado de Saúde – Universidade Federal de Mato Grosso do Sul (UFMS), Campo Grande, MS – Brasil

**Keywords:** Cardiomiopatias, Doença de Chagas, Inflamação, Estado Funcional, Exercício Físico, Imagem de Perfusão do Miocárdio

A cardiomiopatia chagásica crônica resulta da infecção pelo Trypanosoma *cruzi*, transmitido por insetos triatomíneos. A doença, endêmica na América Latina, acomete milhões de indivíduos no mundo. Nas últimas décadas, o aumento migratório elevou o número de pessoas infectadas em regiões não endêmicas como Europa e Estados Unidos. A doença de Chagas permanece como doença negligenciada, caracterizada pela dificuldade para o diagnóstico precoce e patogênese ainda pouco entendida.^1^

O *T. cruzi* infecta cardiomiócitos, células imunitárias e fibroblastos, ativando resposta inflamatória que pode reduzir a carga parasitária e causar a morte de células infectadas. O parasita induz a produção de quimiocinas e citocinas que ampliam a capacidade microbicida de macrófagos e estimulam as sintases de óxido nítrico e a produção de espécies reativas de oxigênio. Linfócitos T CD4 e CD8 também participam da resposta inflamatória com produção de IFN-γ e degranulação de linfócitos T citotóxicos que causam lise de células comprometidas.^2^

Embora a resposta inflamatória inicial seja protetora, sua perpetuação e amplificação levam ao aumento progressivo da injúria miocárdica e remodelamento cardíaco adverso. O quadro agudo é seguido por processo reparativo com ativação de macrófagos do tipo M2, produção de interleucinas anti-inflamatórias e de citocinas da família TGF-β, que induzem diferenciação de fibroblastos e formação de matriz extracelular.^3^ Clinicamente, pacientes com cardiopatia chagásica crônica podem apresentar arritmias, disfunção ventricular e insuficiência cardíaca.

A prática de exercícios físicos é recomendada para mitigar a remodelação e insuficiência cardíaca de diferentes etiologias.^4,5^ Efeitos benéficos têm sido descritos na cardiopatia chagásica crônica como melhora no consumo máximo de oxigênio, fração de ejeção do ventrículo esquerdo (VE), força muscular respiratória, função microvascular e qualidade de vida.^6,7^ Em estudos experimentais, o exercício físico, realizado antes ou após infecção aguda pelo *T. cruzi*, modulou a reação inflamatória, melhorou a resistência ao *T. cruzi*, e reduziu a concentração sérica de creatina quinase miocárdica e a fibrose miocárdica.^8,9^ Na doença crônica, o exercício aeróbio de baixa intensidade melhorou parâmetros morfológicas miocárdicos.^10^

Na edição atual dos ABC Cardiol, o estudo de Damasceno et al.^11^ revelou dados interessantes sobre efeitos do treinamento físico aeróbico na perfusão miocárdica e variáveis morfológicas e funcionais do VE em hamsters com cardiomiopatia chagásica crônica. Alteração da perfusão miocárdica se intensificou com o tempo e foi associada a redução da fração de ejeção e aumento da inflamação miocárdica e da expressão do colágeno tipo I. Redução da área de secção transversa do músculo gastrocnêmio, caracterizando atrofia muscular, também foi observada. O exercício aeróbico atenuou a disfunção sistólica do VE, alterações da perfusão, inflamação miocárdica e atrofia muscular esquelética. Conforme salientado pelos autores, a perfusão miocárdica foi avaliada apenas em repouso. Análise da perfusão em situações de estresse poderia trazer informações valiosas.

Os efeitos do treinamento físico na cardiomiopatia chagásica ainda têm sido pouco explorados. Estudos analisando a influência de diferentes modalidades de exercício e os mecanismos moleculares envolvidos em seus efeitos benéficos serão importantes para o tratamento da cardiomiopatia chagásica crônica.
