# Supplementary material for: Cardiomiopatia Chagásica Crônica: Influência do Exercício Físico
Source: Arq Bras Cardiol. 2024 Sep 18;121(8):e20240507. [Article in Portuguese] doi: 10.36660/abc.20240507 (PMC11495809; doi:10.36660/abc.20240507)
Supplement: Translation

# Translation: Chronic Chagasic Cardiomyopathy: Influence of Physical Exercise

**DOI:** 10.36660/abc.20240507i

**Authors:** Gustavo Augusto Ferreira Mota, Mariana Gatto, Vitor Loureiro da Silva, Paula Felippe Martinez, Marina Politi Okoshi

**Affiliations:** 1 São Paulo SP Brazil Departamento de Clínica Médica – Faculdade de Medicina de Botucatu – Universidade Estadual Paulista (UNESP),1 São Paulo, SP – Brazil; 2 Campo Grande MS Brazil Instituto Integrado de Saúde – Universidade Federal de Mato Grosso do Sul (UFMS), Campo Grande, MS – Brazil

**Keywords:** Cardiomyopathies, Chagas Disease, Inflammation, Functional Status, Exercise, Myocardial Perfusion Imaging

Chronic Chagas cardiomyopathy results from infection with *Trypanosoma cruzi*, transmitted by triatomine insects. The disease, endemic in Latin America, affects millions of individuals worldwide. In recent decades, the increase in migration has elevated the number of infected people in non-endemic regions from Europe and the United States. Chagas disease remains a neglected disease, characterized by difficulty in early diagnosis and poorly understood pathogenesis.^1^

*T. cruzi* infects cardiomyocytes, immune cells, and fibroblasts, activating an inflammatory response that can reduce the parasite load and cause the death of infected cells. The parasite induces the production of chemokines and cytokines that increase the microbicidal capacity of macrophages and stimulate nitric oxide synthases and the production of reactive oxygen species. CD4 and CD8 T lymphocytes also participate in the inflammatory response with the production of IFN-γ and degranulation of cytotoxic T lymphocytes that lysis compromised cells.^2^

Although the initial inflammatory response is protective, its perpetuation and amplification lead to a progressive increase in myocardial injury and adverse cardiac remodeling. The acute condition is followed by a reparative process with activation of M2-type macrophages and production of anti-inflammatory interleukins and cytokines from the TGF-β family, which induce fibroblast differentiation and extracellular matrix formation.^3^ Clinically, patients with chronic chagasic heart disease may present arrhythmias, ventricular dysfunction, and heart failure.

The practice of physical exercise is recommended to improve cardiac remodeling and heart failure from different etiologies.^4,5^ Beneficial effects have been described in chronic chagasic cardiomyopathy, such as improvement in maximal oxygen consumption, left ventricular (LV) ejection fraction, respiratory muscle strength, microvascular function, and quality of life.^6,7^ In experimental studies, physical exercise, performed before or after acute *T. cruzi* infection, modulated inflammatory reaction, improved resistance to *T. cruzi*, and reduced serum concentration of myocardial creatine kinase and myocardial fibrosis.^8,9^ In chronic disease, low-intensity aerobic exercise improved myocardial morphological parameters.^10^

In the current issue of ABC Cardiol, the study by Damasceno et al.^11^ revealed interesting data on the effects of aerobic physical training on myocardial perfusion and LV morphological and functional variables in Syrian hamsters with chronic Chagas cardiomyopathy. Myocardial perfusion defects worsened over time and were associated with reduced LV ejection fraction, increased myocardial inflammation, and Type I collagen expression. A reduction in the gastrocnemius cross-sectional area, characterizing skeletal muscle atrophy, was also observed. Aerobic training attenuated LV systolic dysfunction, perfusion changes, myocardial inflammation, and skeletal muscle atrophy. As pointed out by the authors, only resting perfusion defects were analyzed in the study. Evaluation of perfusion changes under stress situations could bring valuable information.

The effect of physical exercise on Chagas cardiomyopathy is still an open research area. Future studies analyzing the influence of different modalities of exercise and the molecular mechanisms involved in the beneficial effects of exercise on chronic Chagas cardiomyopathy will shed more light on this issue.
